# Predicting Knee Osteoarthritis

**DOI:** 10.1007/s10439-015-1393-5

**Published:** 2015-07-24

**Authors:** Bruce S. Gardiner, Francis G. Woodhouse, Thor F. Besier, Alan J. Grodzinsky, David G. Lloyd, Lihai Zhang, David W. Smith

**Affiliations:** School of Engineering and Information Technology, Murdoch University, Perth, WA Australia; Faculty of Engineering, Computing and Mathematics, The University of Western Australia, Perth, WA Australia; Department of Engineering Science, Auckland Bioengineering Institute, The University of Auckland, Auckland, New Zealand; Departments of Biological Engineering, Electrical Engineering and Computer Science & Mechanical Engineering, Massachusetts Institute of Technology, Cambridge, MA USA; Centre for Musculoskeletal Research, Griffith Health Institute, Griffith University, Gold Coast, QLD Australia; Department of Infrastructure Engineering, The University of Melbourne, Melbourne, VIC Australia

**Keywords:** Biomechanical modeling, Subject-specific risk prediction, Cartilage degeneration, Structural reliability analysis, Extracellular matrix

## Abstract

Treatment options for osteoarthritis (OA) beyond pain relief or total knee replacement are very limited. Because of this, attention has shifted to identifying which factors increase the risk of OA in vulnerable populations in order to be able to give recommendations to delay disease onset or to slow disease progression. The gold standard is then to use principles of risk management, first to provide subject-specific estimates of risk and then to find ways of reducing that risk. Population studies of OA risk based on statistical associations do not provide such individually tailored information. Here we argue that mechanistic models of cartilage tissue maintenance and damage coupled to statistical models incorporating model uncertainty, united within the framework of structural reliability analysis, provide an avenue for bridging the disciplines of epidemiology, cell biology, genetics and biomechanics. Such models promise subject-specific OA risk assessment and personalized strategies for mitigating or even avoiding OA. We illustrate the proposed approach with a simple model of cartilage extracellular matrix synthesis and loss regulated by daily physical activity.

## Introduction

Osteoarthritis (OA) is not easy to define, predict or treat.^31^ Despite extensive research costing many billions of dollars, no drugs have been proven to modify the biological progression of OA, and only a few treatments are proven to relieve symptoms beyond the placebo effect.^38^

Given this failure to find an effective post-diagnosis treatment, perhaps attention should turn to preventing or delaying the onset of cartilage degeneration.^31^ Unfortunately this too is problematic. Except in the particular cases of OA following traumatic injury such as ACL or meniscal damage,^5^,^15^,^45^,^58^ there are many potential interacting causes of OA in an individual. So-called ‘conservative management’ methods (such as planned exercise programs) target subpopulations either at risk of developing OA or rapidly progressing to surgical interventions, such as total knee replacements,^41^ but to be fully effective these methods rely on accurate prediction of susceptible groups.

To date, OA prediction has largely been driven by epidemiological studies that associate risk factors with the likelihood of developing OA.^9^,^12^,^18^,^20^,^27^,^28^,^40^,^54^,^56^,^57^,^64^ A few risk factors recur: for knee OA, these are age, high BMI, low physical activity, high physical activity, muscle weakness, previous injury/surgery (ACL injury and reconstruction, meniscal damage and partial meniscus removal), gender and depression.^2^,^3^,^5^,^12^,^47^,^68^ Genetic pre-disposition is also important,^28^ but this is currently difficult to measure clinically beyond risk associated with family history and its effect on, for example, skeletal anatomy.

Population studies are valuable for long-term healthcare resource planning and for providing general advice about the risk of developing OA. However, this does not translate into patient-specific estimates of the relative or absolute risk of developing OA. By providing personalized risk estimates, people could be motivated to change their modifiable risk factors or to alter decisions when planning the future. This could include making informed decisions about their housing (e.g., avoid stairs and steep terrain), occupation (e.g., avoid heavy manual work), lifestyle (e.g., ensure adequate nutrition) and recreational activities (e.g., avoid certain sports). Patient-specific prediction may also prove important when deciding about future surgery, since implant revisions become more likely with increasing time post-surgery, so the usual advice is to delay joint replacement as long as possible. For these reasons, accurate and timely patient-specific risk prediction is a highly attractive goal.

In this paper, we present what we believe is the most promising and rational approach to realizing this goal: developing patient-specific computational models of the physiological systems related to OA onset and progression, combined with data on population statistical variability. Unlike purely statistical studies, computational modeling allows us to systematically integrate both environmental and genetic patient-specific information into a single model. By integrating diverse sources of information in their biological context, a computational model can transform unexplained variability into explained variability, thereby enabling accurate OA risk predictions.

Which kinds of models will be most effective in turning unexplained variability into explained variability? In the following, we first attempt to quantify the fractions of disease incidence that are due to environmental and genetic (including epigenetic) factors. This helps us decide what kind of model might be most useful in reducing uncertainty in patient-specific risk prediction. In turn, this enables us to estimate what may be achieved using computational models that focus on environmental inputs to drive physiologically-based models, rather than models that focus on genetic factors as inputs (which are harder to quantify). We then illustrate our approach to patient-specific OA prediction using a simple model that is based on known physiology of cartilage tissue. In advocating this approach we also describe an analogous approach from engineering design, called structural reliability analysis. We argue that assessing risk and modes of failure is an appropriate intellectual framework for understanding cartilage OA risk both for an individual and across sub-populations.

## Population Variability: Environmental and Genetic Factors

Ageing is one of the strongest risk factors for OA. For example, the incidence of radiographic OA in the Framingham study was 19% for those over 45 years old, while in the NHANES (III) survey it was 37% for those over 60.^63^ Predictions can be refined beyond age alone by stratifying a population according to one or more criteria and finding the relative risk between the strata at a particular age. Using this approach, obesity emerges as another strong risk factor: compared to a baseline body mass index (BMI) of 22.5, the risk of developing OA increases 1.6 times at a BMI of 25, 3.6 times at a BMI of 30, and 7.5 times at a BMI of 35.^68^ Other risk factors identified include walking patterns, muscle mass, activity levels, occupation (e.g., heavy manual labor and particularly those occupations involving carrying heavy loads, stair-climbing, squatting and kneeling), history of joint injury, history of previous joint surgery, family history of OA, genetic factors (including anatomical variations of the musculoskeletal system), depression and gender.^9^,^17^,^20^,^24^,^27^,^29^,^30^,^43^,^55^,^61^

Studies on twins reveal that environmental risk factors account for between 40 and 60% of OA incidence, with the remaining 60–40% of complementary risk put down to genetic inheritance.^50^,^57^,^61^ There are wide bounds on these estimates because there is a strong interaction between environmental stressors and an organism’s genetically ordained capability to respond to these stressors, which can be difficult to quantify by population studies. Only recently has epigenetics been shown to play a potentially important role;^67^ this further confounds the clear separation of genetic and environmental factors, as epigenetics are not only influenced by ancestors but may also change over a person’s lifetime. However, if environmental risk factors are deemed modifiable, then perhaps as much as 60% of OA may be modifiable or even avoidable. This suggests that a model focusing on environmental risk factors may be both feasible and useful. Presumably an even greater percentage of the population may be able to delay the onset of OA if provided with appropriate advice. Even if one-third of this upper bound estimate can be realized in practice, a 20% reduction in OA and a greater percentage with delayed onset would represent a significant contribution to public health.

In general, excluding autoimmune or other diseases resulting from system dysregulation, most disease states are only realized when environmental conditions stress an organism, or more precisely an organ or tissue within that organism, beyond its repair capabilities. If this capability is chronically exceeded, tissue function deteriorates and the tissue inevitably progresses towards a diseased (i.e., pathological) state. Well after unsustainable processes have commenced, it is eventually recognizable clinically as a chronic disease (in our case, OA). Closer examination reveals that many of the known risk factors for knee OA relate to the mechanical environment experienced by the various joint tissues. For example, since cartilage already faces compressive stresses up to 10 MPa or even 20 MPa^1^ which would quickly obliterate any other soft tissue, it is unsurprising that additional mechanical stressors have adverse consequences.^23^,^60^ In fact, large loads can both cause degradation^6^,^22^,^34^,^36^,^53^ and stimulate repair,^10^,^11^,^22^ implying that the mechanical environment controls a delicate balance between damage and repair.

Importantly, many of these mechanical risk factors are clinically modifiable.^21^ For this reason we have been motivated to develop biomechanical models of cartilage based on tissue turnover of extracellular matrix (ECM) components. The computational model developed later in this paper explicitly incorporates damage and repair processes for three key matrix components, albeit at a high level omitting the specifics of the processes involved. From the model output an assessment can be made of the tissue’s sustainability for a variety of environmental loadings, from which predictions can be made about the likelihood of an individual developing OA. In the next section we discuss cartilage ECM and its damage and repair, before applying this in a first generation patient-specific risk prediction model based on cartilage degeneration.

## Cartilage Extracellular Matrix and Mechanical Failure

**Figure 1:**
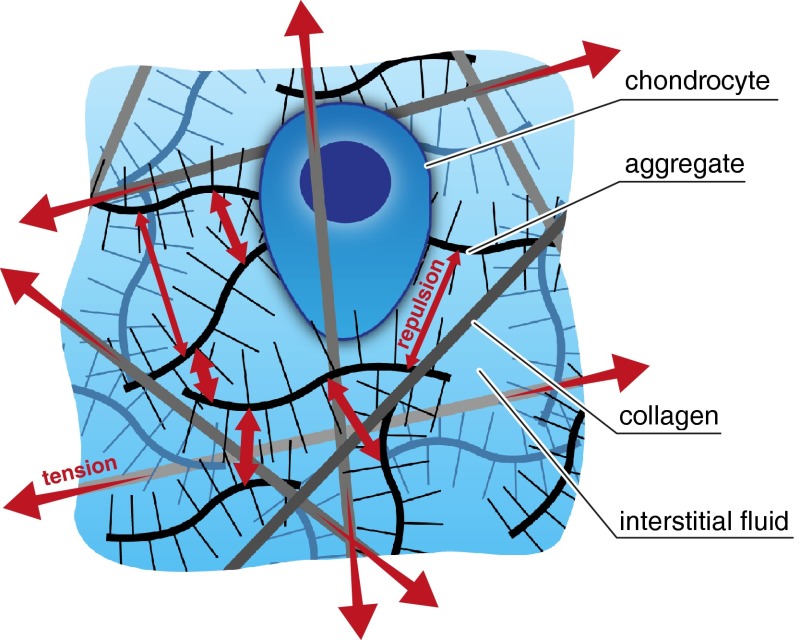
Aggrecan, produced by chondrocytes, carries a strong negative charge. The resulting repulsion (electrical and osmotic, represented by the small red arrows) gives cartilage a tendency to swell.^22^ The collagen network within the cartilage (anchored to the underlying bone) provides cartilage with tensile strength and constrains the swelling and release of aggrecan to the joint space.^22^ The collagen is therefore normally under tension (large red arrows). Illustration not to scale.

The ECM of cartilage includes dozens of collagens, proteoglycans, and glycoproteins,^25^ all enmeshed within intratissue water, called the interstitial fluid (Fig. 1). Arguably the two most structurally important macromolecules that regulate the tissue’s biomechanical functional properties are type II collagen and the proteoglycan aggrecan (as aggregate). The stiffness of cartilage under compression comes from both the repulsion between negatively charged aggrecans and the difficulty that the fluid has in squeezing out of the tissue.^22^,^49^ The interstitial fluid leaving the tissue then helps to give cartilage its famously low frictional properties *via* so-called mixed-mode lubrication.^42^ In addition, collagen helps resist shear loads and the loss of aggrecan itself, which otherwise would swell apart and be rapidly lost from cartilage.^49^

In the clinical literature there is some discussion as to whether to define OA by clinical symptoms, like pain and disability, or by structural changes inferred through radiology or MRI.^16^,^26^ We take a more function-oriented approach and consider OA as an inability of cartilage to maintain its functional mechanical properties: the tissue has failed when fundamental mechanical variables, such as deformational resilience and interstitial fluid pressure, fall below levels required to maintain tissue integrity. Note that the root cause of this failure may be internal or external to the cartilage tissue; indeed, OA is commonly regarded as a disease of the whole joint.^37^ Our functional definition of OA is consistent with that advocated by the Osteoarthritis Research Society International (OARSI) for early identification of OA risk and progression.^31^

**Figure 2:**
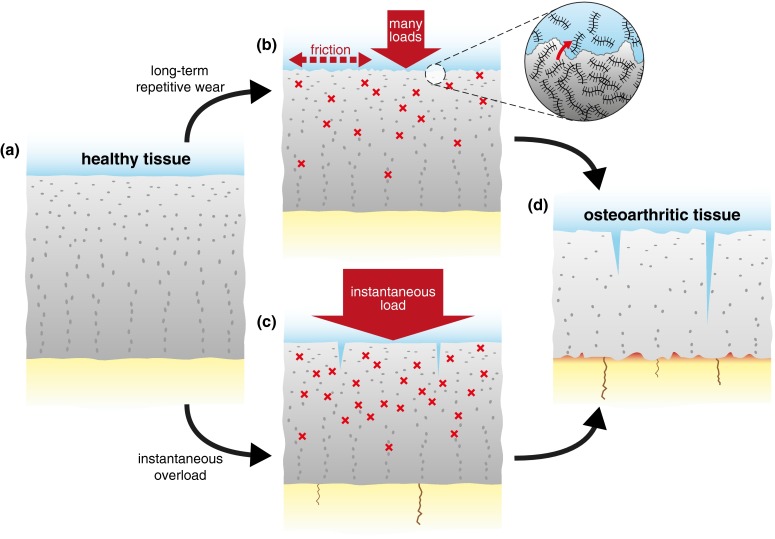
Illustration of two potential mechanically initiated failure pathways to OA. Note other pathways (not shown), either mechanical or non-mechanical, may also initiate OA. (a) Normal healthy cartilage may experience (b) long-term overuse or repetitive small loads, which causes wear at the cartilage surface and exposes chondrocytes to high strains by the resulting consolidation under cyclic loading. (c) Alternatively, healthy cartilage may experience a high impact (short-term) load leading to splits, chondrocyte death, cytokine release with protease-mediated ECM degradation, and damage to the subchondral bone. (d) Ultimately, both routes result in failure as the cartilage repair capacity is exceeded.

Although chondrocytes are known to adjust the ECM in response to chemical and environmental signals,^10^,^22^ substantial and/or long-term changes in these signals make the tissue more vulnerable to failure. This can occur through various mechanisms (exemplified in Fig. 2). Excessive tissue deformation, from either abnormally large sustained loads or abnormally weak tissue, can cause chondrocyte apoptosis.^36^ Insufficient lubrication between contacting cartilage surfaces or excessive activity will lead to excessive cartilage wear^33^ (as experienced by plumbers^14^ or cross-country skiers,^44^ for example). On the other hand, too low activity or static loads are known to inhibit ECM repair by retarding chondrocyte synthesis of aggrecan and collagen.

If we are to understand the biomechanical factors contributing to tissue failure, or OA, we need to start by understanding how observable differences in joint loads and geometry translate to changes in the mechanical environment experienced by the cartilage itself. More generally, since OA is rarely—if ever—the result of mechanics alone, a so-called mechanistic model of cartilage homeostasis is needed.

## Mechanistic Models vs. Statistical Models

A mechanistic model is one pertaining to the underlying physical, chemical and biological mechanisms, describing how these processes interact and evolve in time. Far from being limited to mechanical loading alone, such a model could also involve cell signaling pathways, metabolic effects, ECM synthesis and proteolysis, and so on. Unlike a purely statistical approach, an appropriate mechanistic model allows experimental data to be placed in its proper context. For example, the interaction of the insulin-like growth factor IGF-1 with the binding proteins and proteases found in serum, synovial fluid and cartilage only makes sense when it is placed in the context of diffusive transport into the tissue and the ability of the tissue to regulate its exposure.^62^,^66^ BMI furnishes a simpler example: we know it is statistically connected to OA risk, but whether or not this is due to mechanical reasons can only be ascertained in a subject-specific model of cartilage mechanics incorporating knee geometry and the equations of mass and momentum balance. Furthermore, a mechanistic model enables ‘*in silico*’ experiments to investigate disease processes or reveal treatment strategies based on an individual’s combination of ‘parameters’.

The above somewhat rosy view of mechanistic modeling is undermined by imperfect knowledge of model structure and parameter values. The art of modeling is intuiting a model structure that can give insight into the question being asked. For example, we have argued above that focusing on turnover of the ECM is an appropriate conceptual starting point for questions related to OA prediction. Others might choose a different model structure (e.g., focus on joint forces). There is no single right way to model OA mechanistically, and model structure will vary with the modelers and the specific question being addressed.

It is useful here to treat model uncertainty as belonging to two main types. Imagine a model for a generic person. Many of the model parameters and even the core structure of the model, such as the phenomena it includes, will be only known to within a range; we call this population uncertainty. In contrast to population uncertainty, we refer to individual uncertainty as how a particular individual may vary from this generic person. Whenever uncertainty arises, stochastic approaches need to be coupled to mechanistic models.

**Figure 3:**
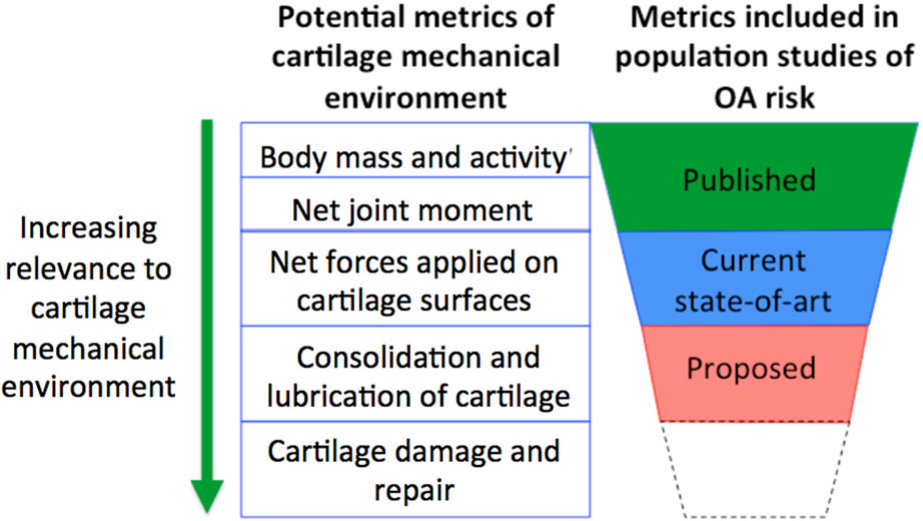
Conceptualizing cartilage mechanical environment metrics that incorporate more known factors.

**Figure 4:**
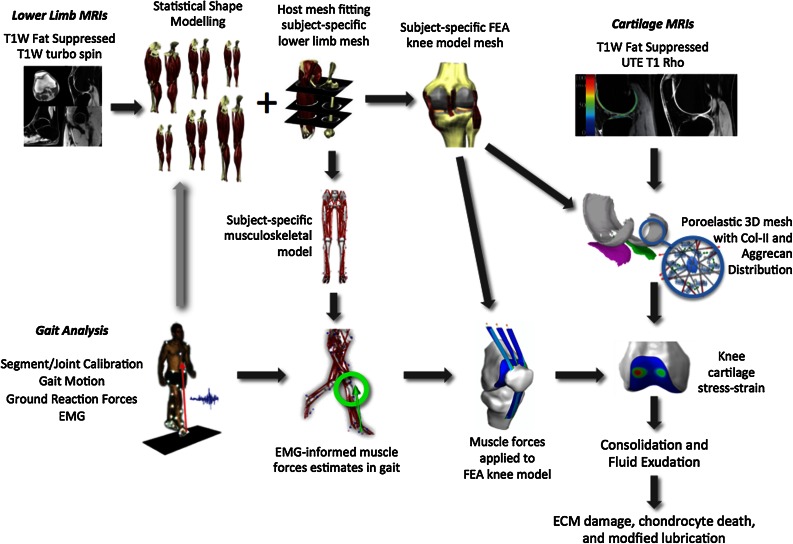
Workflow for integrating imaging, gait and cartilage quality data into a multiscale subject-specific model of human knee cartilage. For further discussion on each component, see host-mesh fitting,^19^ EMG-informed muscle forces in gait,^35^ knee cartilage stress–strain,^7^,^8^ and poroelastic models of cartilage.^49^,^59^,^65^ We argue that tissue-level metrics of cartilage consolidation and fluid exudation will have a stronger association with cartilage loss and defect enlargement than risk factors used in previous studies.

Nevertheless, it may be possible to remove some of the unexplained population variability weakening the association between, for example, current biomechanical risk factors and OA outcomes. Indirect mechanical measures of loading of the medial compartment of the knee, such as knee adduction moments, provide a much better prediction for OA progression than body weight or frontal plane knee alignment, either alone or in combination.^46^ Extrapolating, we would expect that if even more relevant biomechanical factors were to be evaluated, such as the duration of lubrication and tissue consolidation, unexplained population variability may be reduced and so our predictive ability would increase. These subject-specific tissue mechanical conditions are likely to be a stronger metric to associate with OA risk than, say, BMI or knee adduction moments (Fig. 3). A multiscale subject-specific modeling approach, as depicted in Fig. 4, may be able to provide these stronger metrics.

## Structural Reliability Analysis

Osteoarthritis can be viewed as a condition in which the ECM components fail to deliver the required mechanical function under the loads experienced. The advantage of expressing the disease problem this way is that we begin to see cartilage as a structure, which has a risk of failure (due to cell death or excessive proteoglycan loss, for example) when subjected to varying and uncertain loads. This allows us to invoke well-established concepts and methods from structural reliability analysis^4^,^51^ to predict OA risk.

**Figure 5:**
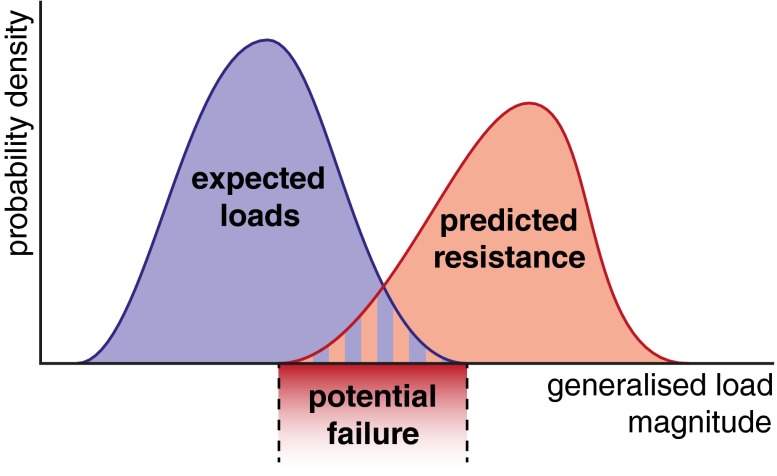
Structural reliability analysis: the risk of failure increases as the distribution of expected loads increasingly overlaps the distribution of the expected ultimate load of the structure (resistance). The load measure need not be a pressure or force, but rather some generalized measure of the duress under which the structure has been placed.

Conceptually, structural reliability analysis is simple. When designing a structure, such as bridge, an engineer chooses particular structural components in order to resist an expected load, such as wind or earthquake. This load is generally not a single value, but rather a distribution of potential values. However, as structures become more complex, variability in the properties of structural components (variation in strut thicknesses, for example) introduces uncertainty into the ability of the structure to resist a given load. The engineer’s task is then to compare the expected loads and the structure’s likely resistance in order to estimate the risk of failure. This is done by estimating a probability density function for the expected loads and another for the structure’s ultimate load, called its resistance. The overlap in these two densities, where the load exceeds the structure’s ability to resist it, then relates to the risk of failure (Fig. 5).

In cartilage we can estimate the distribution of mechanical loads using the subject-specific multiscale approach illustrated in Fig. 4, combined with measures of subject activity levels. The cartilage resistance to this load depends on what would otherwise be regarded as biological processes of ECM synthesis and loss through the action of proteases, mechanical damage and transport through the tissue surface. Non-mechanical challenges, such as inflammatory cytokines or hormonal changes, enter *via* the ability of cartilage ECM to be sufficiently maintained to provide a resistance to the distribution of potential loads. Mechanistic models for each of load and resistance would be used along with the uncertainty in each model variable to create the probability density functions found in Fig. 5.

The terms ‘load’ and ‘resistance’ broaden to ‘generalized loads’ and ‘generalized resistances’ for the potentially many dimensions upon which the mechanical and chemical function of cartilage can be assessed and the many resulting failure modes. In Figs. 3 and 4 we suggested, for example, that the consolidation and fluid exudation rate may lead to increased cartilage damage, *via* increase in cell death, collagen damage and surface wear. We can reframe this loss of fluid exudation under sustained load as a short-term loss of the cartilage to achieve the mechanical function of adequately lubricating the joint, with long-term consequences of excessive cartilage wear and eventual failure of the joint. Alternatively, excessive tissue deformation can lead to an increased rate of cell death, reducing tissue repair capacity in the short term and causing the eventual failure of the tissue to evenly distribute contact loads in the long term. In general, the distributions depicted in Fig. 5 are only snapshots in time that will, in fact, evolve, and OA can be due to changes in the distribution of either the load, or the resistance, or both: an otherwise normal individual may develop OA simply by shifting the load profile, or an individual with activity within the normal range could develop OA because they have a genetic profile such that their ECM is less resistant to mechanical loading.

Although Fig. 5 is useful for depicting the basic concepts, it falls down if there are multiple modes of failure (each requiring its own axis in Fig. 5) or if, as for cartilage, the resistance changes in time through load-dependent damage and repair. In these cases, load and resistance distributions are difficult, if not impossible, to compute directly, and it is instead appropriate to proceed by simulating a large number of realizations of a stochastically loaded mechanistic model. Specifically, in each realization, model parameters are randomly selected from an expected range and the structure is allowed to evolve. For cartilage this process can be considered either equivalent to computationally creating a study population of subjects based on population variability (population uncertainty) or building up potential outcomes for an individual based on a subject’s parameter uncertainty (individual uncertainty). We will exemplify this approach in the following section using a simple but informative model.

## Example of an OA Risk Prediction Model

To illustrate the core principles discussed above, we now construct an elementary mechanistic model of long-term cartilage health for the purposes of OA onset prediction. Although such a model may be too simplistic to produce accurate patient-specific predictions as it stands, it serves as a concept for a modular, updatable model, making explicit the key inputs required from the many investigators involved in cartilage research, such as epidemiologists, cell biologists, geneticists and biomechanical scientists.

## Model Construction

The model tracks the densities of three main cartilage tissue components: chondrocytes, aggrecan and collagen. These are represented by their spatial averages. Let *n*_*t*_ denote the average number density of chondrocyte cells in the cartilage at time *t* (measured in days). Similarly, let *a*_*t*_ denote the average mass density of aggrecan (assumed mostly bound in aggregates), and let *c*_*t*_ denote the average mass density of collagen (assumed mostly structural type II). These are updated over time according to damage and repair rules that depend on randomly generated physical activity.

First, we characterize the mechanical loading of the tissue. Suppose that on day *t*, the subject performs activities amounting to loading the tissue with some stress *σ*_*t*_ at an overall frequency *f*_*t*_ when averaged over the whole day. These are drawn from distributions whose parameters depend on the subject’s daily habits. For simplicity, we assume these distribution parameters are constant in time.

If the activity continues for long enough, then the tissue will consolidate to an equilibrium state. We can form a representative metric of this state by calculating the induced cartilage strain *ε*_*t*_ using a simplified one-dimensional consolidation model. For simplicity we assume the tissue is homogeneous, though a refined model would likely require spatial variance of the cartilage geometry and material parameters.^59^ Now, suppose that the strain *ε*_*t*_ is borne entirely by the aggregates. It is known that aggrecan of density *a* has an osmotic pressure fitting the virial expansion^6^$\Pi \left( a \right) = RT\left( {\alpha_{1} a + \alpha_{2} a^{2} + \alpha_{3} a^{3} } \right)$, with parameter values given in Table 1. However, under a compressive strain $\varepsilon > 0$, the true local aggrecan density is $a/(1 - \varepsilon )$. Therefore at a consolidated equilibrium, balancing the osmotic pressure of compressed aggrecan against the imposed stress *σ*_*t*_ yields the stress–strain relation $\Pi \left( {\frac{{a_{t} }}{{1 - \varepsilon_{t} }}} \right) = \sigma_{t}$. This can then be solved for *ε*_*t*_, the strain at equilibrium.

**Table 1:** Osmotic pressure parameters.^6^

Parameter	Value
*R*	8.3 × 10^3^ mL kPa/mol/K
*T*	300 K
$\alpha_{1}$	1.4 ×10^−7^ mol/mg
$\alpha_{2}$	4.4 × 10^−9^ mol mL/mg^2^
$\alpha_{3}$	5.7 × 10^−11^ mol mL^2^/mg^3^

We now need a loading metric to stimulate the cartilage tissue damage and repair models. The equilibrium strain can be combined with the loading frequency *f*_*t*_ to form the daily activity level $A_{t} = \varepsilon_{t} f_{t}$, a simple metric of how vigorous the day’s activity has been from the viewpoint of the cartilage. This daily activity level will then be an input into the model’s damage and repair processes to calculate the stimulated production and loss of the three cartilage components.

Next, we formulate the damage and repair equations for the chondrocytes *n*_*t*_, aggrecan *a*_*t*_ and collagen *c*_*t*_. Chondrocytes can repair, to some extent, by proliferation; this is noticeable in osteoarthritic conditions, perhaps to replenish chondrocyte loss and increase the ECM repair capacity, though in healthy tissue chondrocyte turnover is low. Conversely, chondrocytes can be driven to apoptosis either by repetition of high load events or by one single extremely high load (traumatic) event, both of which result in a high level of the activity *A*_*t*_. To model these two competing processes, we write

$$n_{t + 1} = n_{t} + \rho^{\left( n \right)} \left( {1 - \frac{{n_{t} }}{{n^{\left( 0 \right)} }}} \right)n_{t} - \lambda^{\left( n \right)} D^{\left( n \right)} \left( {A_{t} } \right)n_{t}$$

where $\rho^{\left( n \right)}$ is a maximal proliferation rate per day up to a healthy number density $n^{\left( 0 \right)}$, and $D^{\left( n \right)} \left( {A_{t} } \right)$ is the chondrocyte damage function rating how deleterious the day’s activity *A*_*t*_ was on a scale from 0 to 1, with $\lambda^{\left( n \right)}$ the maximum fraction of chondrocytes potentially removed per day. The damage function is taken to be a shifted sigmoid function

$$D^{\left( n \right)} \left( {A_{t} } \right) = \frac{{1 + \exp \left( { - \mu^{\left( n \right)} A^{\left( 0 \right)} } \right)}}{{1 + \exp \left( { - \mu^{\left( n \right)} (A_{t} - A^{\left( 0 \right)} )} \right)}} - \frac{{1 + \exp \left( { - \mu^{\left( n \right)} A^{\left( 0 \right)} } \right)}}{{1 + \exp \left( {\mu^{\left( n \right)} A^{\left( 0 \right)} } \right)}}$$

with sigmoid gradient $\mu^{\left( n \right)}$ and threshold position $A^{\left( 0 \right)}$. The latter encodes the onset threshold of tissue-affecting activity levels, and will also appear in other components as a universal threshold; the former encodes the suddenness of damage onset, whose value is chondrocyte-specific.

Aggrecan is synthesized by each chondrocyte at some activity-dependent rate. However, it is also lost through the tissue surface by degradation and pressure-driven advection. This loss rate will increase as the collagen content decreases, because collagen acts to retain aggrecan. We write

$$a_{t + 1} = a_{t} + R^{\left( a \right)} \left( {A_{t} } \right)n_{t} - \left( {\lambda^{{\left( {a,0} \right)}} + \lambda^{{\left( {a,1} \right)}} \exp \left( {1 - \frac{{c_{t} }}{{c^{\left( 0 \right)} }}} \right)} \right)a_{t}$$

where $R^{\left( a \right)} \left( {A_{t} } \right)$ is the activity-dependent synthesis rate per cell, $\lambda^{{\left( {a,0} \right)}}$ is the baseline aggrecan loss rate when $c_{t} = c^{\left( 0 \right)}$, with $c^{\left( 0 \right)}$ the baseline healthy collagen content, and $\lambda^{{\left( {a,1} \right)}}$ is the maximal additional aggrecan loss rate as collagen depletes. The activity-dependent synthesis rate is given by

$$R^{\left( a \right)} \left( {A_{t} } \right) = \rho^{{\left( {a,0} \right)}} \exp \left( { - \frac{{A_{t} }}{{A^{\left( 0 \right)} }}} \right) + \rho^{{\left( {a,1} \right)}} \left( {1 - \exp \left( { - \frac{{A_{t} }}{{A^{\left( 0 \right)} }}} \right)} \right)$$

where $\rho^{{\left( {a,0} \right)}}$ and $\rho^{{\left( {a,1} \right)}}$ are the minimal and maximal synthesis rates per chondrocyte, respectively, and $A^{\left( 0 \right)}$ is as for the chondrocytes.

Collagen is also synthesized by each chondrocyte. On the other hand, the collagen network has a natural rate of loss by proteolytic degradation, and can also be directly damaged through mechanical loading or excessive friction and wear. These processes are encoded as

$$c_{t + 1} = c_{t} + R^{\left( c \right)} \left( {A_{t} } \right)n_{t} - \left( {\lambda^{{\left( {c,0} \right)}} + \lambda^{{\left( {c,1} \right)}} D^{\left( c \right)} \left( {A_{t} } \right)} \right)c_{t}$$

where $\lambda^{{\left( {c,0} \right)}}$ is the baseline loss rate and $\lambda^{{\left( {c,1} \right)}}$ is the maximal daily damage rate. The synthesis rate, identical in form to the aggrecan, is

$$R^{\left( c \right)} \left( {A_{t} } \right) = \rho^{{\left( {c,0} \right)}} \exp \left( { - \frac{{A_{t} }}{{A^{\left( 0 \right)} }}} \right) + \rho^{{\left( {c,1} \right)}} \left( {1 - \exp \left( { - \frac{{A_{t} }}{{A^{\left( 0 \right)} }}} \right)} \right)$$

with new rate coefficients for collagen. The damage function is identical in form to the damage for the chondrocytes, reading

$$D^{\left( c \right)} \left( {A_{t} } \right) = \frac{{1 + \exp \left( { - \mu^{\left( c \right)} A^{\left( 0 \right)} } \right)}}{{1 + \exp \left( { - \mu^{\left( c \right)} (A_{t} - A^{\left( 0 \right)} )} \right)}} - \frac{{1 + \exp \left( { - \mu^{\left( c \right)} A^{\left( 0 \right)} } \right)}}{{1 + \exp \left( {\mu^{\left( c \right)} A^{\left( 0 \right)} } \right)}}$$

again with a new coefficient $\mu^{\left( c \right)}$.

## Model Application to Risk Prediction

A statistical approach is to view some, or all, of the inputs (and/or model parameters) as randomly varying, and the model outputs, on repeated simulation runs, as realizations of an underlying probability distribution for the trajectory of the tissue health over time. This provides a more realistic and more personalizable approach to OA prediction on longer time scales, as both uncertainty in parameter estimation and variability in different patients’ lifestyles can be incorporated readily. From this approach, onset predictions can be estimated once the model is tuned to a particular patient, and, importantly, mitigation strategies can be explored by altering these parameters.

The parameters we will use for this example are given in Table 2. While some variables are at least approximately known, such as cell and collagen densities, others, such as loss rates in response to activity, lack solid quantitative data. We have chosen variables that give plausible results for susceptible patients in order to illustrate the model, on the understanding that future work is necessary to verify and calibrate models like this. Exploring population uncertainty would then correspond to varying these parameters for each simulation run. In this instance, we will hold these parameters constant and instead explore individual uncertainty through randomly varying daily activity. For the distributions of the activity variables *σ*_*t*_ and *f*_*t*_, we choose normal distributions of respective means $\bar{\sigma }$, $\bar{f}$ and respective variances $\left( {\frac{1}{3}\bar{\sigma }} \right)^{2}$, $\left( {\frac{1}{3}\bar{f}} \right)^{2}$. Choosing different values of $\bar{\sigma }$ and $\bar{f}$ then allows us to simulate low-, medium- and high-activity lifestyles.

**Table 2:** Parameter values in OA model. See Ref. 39 for component densities.

Parameter	Value
$\rho^{\left( n \right)}$	0.002/day
$\rho^{{\left( {a,0} \right)}}$	1.1 × 10^−8^ mg/day
$\rho^{(a,1)}$	$\frac{3}{2}\rho^{{\left( {a,0} \right)}}$
$\rho^{{\left( {c,0} \right)}}$	1.1 × 10^−8^ mg/day
$\rho^{{\left( {c,1} \right)}}$	$\frac{3}{2}\rho^{{\left( {c,0} \right)}}$
$n^{\left( 0 \right)}$	10^8^/mL
$\lambda^{\left( n \right)}$	0.01/day
$\lambda^{{\left( {a,0} \right)}}$	0.01/day
$\lambda^{{\left( {a,1} \right)}}$	0.005/day
$c^{\left( 0 \right)}$	170 mg/mL
$\lambda^{{\left( {c,0} \right)}}$	0.006/day
$\lambda^{{\left( {c,1} \right)}}$	0.01/day
*A* ^(0)^	30% × 0.3 Hz
$\mu^{\left( n \right)}$	50 s
$\mu^{\left( c \right)}$	100 s

We use the model to simulate an abrupt change in activity. In this scenario, a person switches lifestyle from ‘normal impact’ daily loading to either ‘high impact’ or ‘low impact’ loading distributions, characterized by adjusting the distribution parameters $\bar{\sigma }$, $\bar{f}$. The normal impact loading is at a level permitting healthy tissue homeostasis, whereas the others are potentially injurious regimes: high impact represents overload damage (e.g., through obesity or abnormal activities), and low impact represents under-synthesis (e.g., through too sedentary a lifestyle).

To characterize the overall health of the cartilage at every point in time, we define the tissue health as the difference of consolidated strain from 35% under a 400 kPa test load, with 35% chosen as a typical tolerable maximal tissue strain. A strain greater than 35% under the test load then translates into a negative health metric and therefore indicates potential OA onset. One could also construct a health surrogate based on regenerative capacity, say, to highlight longer-term regimes of OA danger.

**Figure 6:**
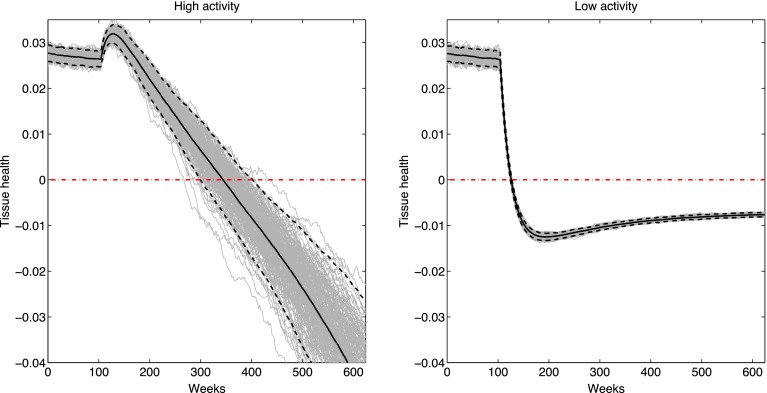
Cartilage health (as defined in the text) during an abrupt shift from medium to high activity (left) or from medium to low activity (right). Grey lines are individual activity realizations; solid black lines are the means over all realizations, with 95% confidence intervals in dashed black lines. Red dash-dotted line indicates the zero health OA danger threshold.

Figure 6 shows the effects of switching from medium activity to high activity or low activity. After a stable period at $\bar{\sigma } = 350 \;{\text{kPa}}$ and $\bar{f} = 0.1\; {\text{Hz}}$, the activity distribution is abruptly switched to either $\bar{\sigma } = 450\; {\text{kPa}}$ and $\bar{f} = 0.12 \;{\text{Hz}}$ (high activity) or $\bar{\sigma } = 200 \;{\text{kPa}}$ and $\bar{f} = 0.02 \;{\text{Hz}}$ (low activity). In high activity case, an initial rise in tissue health from increased activity-driven synthesis is soon outweighed by the long-term effects of damage leading to a slow but persistent decline in tissue health. In the low activity case, the decrease in activity-driven synthesis is sufficient to quickly drop the tissue below the indicated danger threshold; though it does not keep decreasing like in the high activity case, the tissue is now more susceptible to sudden impact loading and may be at increased risk of age-related OA. These two circumstances correspond to respectively shifting either the load or resistance curves in Fig. 5, as discussed earlier.

**Figure 7:**
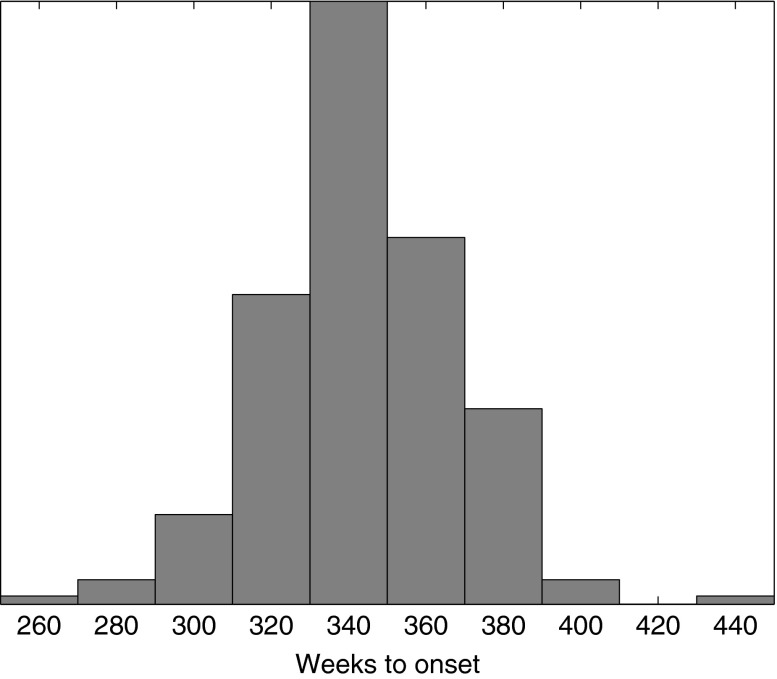
Distribution of OA danger threshold hitting times in the high activity example of Fig. 6.

The high activity switch exhibits a range of different potential OA onset thresholds depending on the particular realizations of the daily activity distribution. This information can be best presented to a patient through statistics of the distribution of OA onset hitting times; that is, the first time at which a particular trajectory crosses the zero health axis. For our example data, this distribution is given in Fig. 7, which predicts an OA onset time of 345 ± 47 weeks.

## Conclusions

We have argued that combining mechanistic computational models with statistical approaches under the umbrella of structural reliability analysis provides a promising framework for overcoming the current challenges in providing subject specific recommendations for avoiding OA onset and conservatively managing OA progression. Although multiscale subject-specific models are likely needed to encompass more of the salient characteristics that OA patients may present with, the example model presented here does develop tissue changes that may well represent the OA cartilage degradation process. We believe that by using such models, stronger OA patient-specific risks will be found if direct metrics for the tissue mechanical environmental stressors, such as consolidation and fluid exudation, rather than indirect measures like BMI and physical activity, are used.

While reliable patient-specific predictions are not yet possible, how quickly they will emerge depends mainly on the speed with which high quality patient-specific data becomes available at an affordable price. At present, getting sufficient information to feed into a mechanistic model is a challenge. However, technology is evolving rapidly. With the ongoing developments in high-throughput genomic and proteomic technologies and imaging technologies (including computer vision of gait), alongside musculoskeletal data obtained from gait laboratories and activity monitors in mobile phones, data to drive patient-specific models may become available sooner than one may think. Indeed, high quality complete genome sequencing can now be accomplished for less than one thousand dollars, proteomic analysis is developing rapidly, and it is now possible to analyze the blood and synovial fluid to better understand inflammatory drivers of OA.^32^ Furthermore, MRI imaging can now quantitate damage to the collagen network following joint trauma and track collagen network recovery over a number of years.^13^

Model development and validation will likely be both iterative and opportunistic. It will be iterative in the sense of a Bayesian approach: a new data set is first used for validation, then folded into the model calibration by updating model parameters, so the model is always improving with each data cycle. On the other hand, development will be opportunistic in the sense that when a new technology arises, such as phone applications that faithfully record a person’s activity levels, then improvements in this aspect of the model may be driven ahead of others. New data is arriving all the time from population and lab based studies, as well as community wide projects such as the Knee Osteoarthritis Initiative. We can dream of a time when, in contrast to these relatively uncoordinated data collections, the OA community starts collecting data specifically to inform a model. This is beginning to occur in the study of other diseases.^48^,^52^

Finally, it is important to remember that when people refer to patient-specific models or risk predictions, it is not expected that everything is known about the individual. A compromise must always be made as to what data can be obtained, and at what financial cost and patient inconvenience. The question should then be: what data is most informative about the risk of OA amongst that which can be reasonably measured? In such an approach all other unknown variables would be assumed to be either at the population average, or better sampled randomly from an assumed population distribution. Naturally, the clinical utility of this approach rests on whether or not the obtainable data yields a risk assessment more accurate than that already known for population risk as a whole. This is yet to be seen. However, there is utility beyond immediate clinical application. As with bridge designers, simply putting the risk assessment into this mechanistic-statistical framework of structural reliability analysis helps to define the problem, allowing us to identify new and efficient strategies to minimize the risk of failure.
